# *Rhizopus oligosporus* and *Lactobacillus plantarum* Co-Fermentation as a Tool for Increasing the Antioxidant Potential of Grass Pea and Flaxseed Oil-Cake Tempe

**DOI:** 10.3390/molecules25204759

**Published:** 2020-10-16

**Authors:** Bożena Stodolak, Anna Starzyńska-Janiszewska, Magdalena Mika, Agnieszka Wikiera

**Affiliations:** Department of Biotechnology and General Technology of Food, Faculty of Food Technology, University of Agriculture in Krakow, 30-149 Krakow, Poland; anna.starzynska@urk.edu.pl (A.S.-J.); magdalena.mika@urk.edu.pl (M.M.); agnieszka.wikiera@urk.edu.pl (A.W.)

**Keywords:** antiradical activity, total phenols, water-soluble fraction, co-fermentation, flaxseed oil-cake, grass pea tempe

## Abstract

Tempe-type fermentation originating from Indonesia can enhance the antioxidant activity of plant material. However, this biological potential depends on substrates and applied microorganisms. This study aimed to determine whether co-fermentation with *Rhizopus oligosporus* and *Lactobacillus plantarum* improved antioxidant activity of tempe obtained from grass pea seeds with flaxseed oil-cake addition (up to 30%). For this purpose, substances reacting with Folin–Ciocalteu reagent and free radicals scavenging potential were measured in water-soluble fractions and dialysates from simulated in vitro digestion. Additionally, the water-soluble phenolic profile was estimated. The higher level of water-extractable compounds with antioxidant activity was determined in co-fermentation products than in fungal fermentation products. Moreover, the fermentation process with the use of *L. plantarum* contributed to a greater accumulation of some phenolic acids (gallic acid, protocatechuic acid) in tempe without having a negative effect on the levels of other phenolic compounds determined in fungal fermented tempe. During in vitro digestion simulating the human digestive tract, more antioxidant compounds were released from products obtained after co-fermentation than fungal fermentation. An addition of 20% flaxseed oil-cake and the application of bacterial–fungal co-fermentation, can be considered as an alternative tool to enhance the antioxidant parameters of grass pea tempe.

## 1. Introduction

Tempe is a food product of Indonesian origin obtained from legumes (mainly soy), cereals or agricultural waste via solid state fermentation. The indispensable microorganism in tempe-type fermentation is *Rhizopus* sp., usually *R. oligosporus* [1]. Tempe production can be regarded as an alternative way of processing legume seeds to obtain convenient foods of diversified nutritional, organoleptic and bioactive properties. An interesting example is grass pea (*Lathyrus sativus* L.) seeds, which contain valuable proteins and a relatively high level of mineral compounds. Grass pea seeds from European countries are considered a good source of phenols in a vegetarian diet, with dominant *p*-coumaric acid derivatives in the phenolic profile [2]. Moreover, fermentation of grass pea seeds with *Rhizopus* strains was proven to significantly enhance the total phenol level and antioxidant potential of the substrate [3].

The inclusion of flaxseed oil-cake as a co-substrate in grass pea-based tempe creates additional opportunities to enrich the nutritional and bioactive potential of the product. Due to its high nutritional value, flaxseed oil-cake obtained after cold press oil extraction can be used as a food ingredient. The introduction of flaxseed or flaxseed processing by-products to various plant-derived matrices usually enriches the material in phenols and enhances its antioxidant potential, as proven in the case of bread [4] and grass pea tempe [5]. Flaxseed is the richest source of lignans, mainly secoisolariciresinol (SECO) and its glucoside SDG (secoisolariciresinol diglucoside) of proved antioxidant activities [6].

Though the tempe-type processing can be considered a relatively simple fermentation technique, the selection of the microorganism and the optimal conditions for its growth are indispensable for obtaining products of favourable properties. When alternative substrates such as agricultural by-products are included, an advantageous option can be to introduce into the inoculum a microorganism whose activity would support the growth and activity of the *Rhizopus* strain. Lactic acid bacteria (LAB) derived from seeds or microbial starters can take part in the tempe fermentation process and contribute to the suppression of spoilage microflora, and thus prolong the shelf life of tempe [1]. Moreover, simultaneous growth of *R. oligosporus* and *L. plantarum* on common beans was proven to result in tempe products of improved nutritional composition as well as diversified antioxidant activity [7]. Lactic acid bacteria (LAB) are commonly applied in food fermentations, and *Lactobacillus plantarum* is most frequently used for plant materials [8].

The purpose of the experiment was to determine whether the presence of LAB during the tempe-type fermentation of grass pea seeds with flaxseed oil-cake addition allows obtaining a product with more advantageous antioxidant parameters than the process carried out only with the use of mould. Different grass pea and flaxseed oil-cake mixtures were tested in order to obtain the combination that ensures obtaining a product with the highest bioactive potential.

## 2. Results and Discussion

### 2.1. Antioxidant Activity

#### 2.1.1. Substances Reacting with Folin–Ciocalteu Reagent

The level of compounds reacting with Folin–Ciocalteu reagent (FCRS) depended on the substrate kind—the addition of 10% (FOC10) and 20% (FOC20) flaxseed oil-cake to grass pea seeds resulted in an increase in FCRS by 67% and 87%, respectively, whereas the highest dose (30%) of oil-cake (FOC30) did not further enrich the tempe in the said compounds (Table 1). The fermentation method also had a significant impact on the FCRS levels. In the fungal fermentation products, around 115% more FCRS was determined than in the respective substrates. The application of fungal–bacterial inoculum resulted in a small, though significant (by 5%), increase in FCRS, as compared to the material fermented solely with mould. The highest levels of FCRS were measured after the bioprocessing of FOC20 and exceeded the values reported for fermented and co-fermented FOC0 by 78% and 92%, respectively. Substances that react with Folin–Ciocalteu reagent are mainly phenolic compounds. Therefore, the increase in FCRS observed after fermentation was most likely the result of the release of phenols from their glycosides or the enhanced solubility of phenols due to the action of microbial enzymes. The release of phenols has often been correlated with increased activity of β-glucosidase, but also α-amylase and xylanase [9,10]. The influence of LAB on the accumulation of soluble phenols could be of two types: (i) indirect, because bacteria could support the development of mycelium, therefore contributing to the greater activity of fungal β-glucosidases, and (ii) direct, as *L. plantarum* could also synthesize the aforementioned enzymes [11,12].

#### 2.1.2. ABTS^+•^ Scavenging Activity

**Figure 1:**
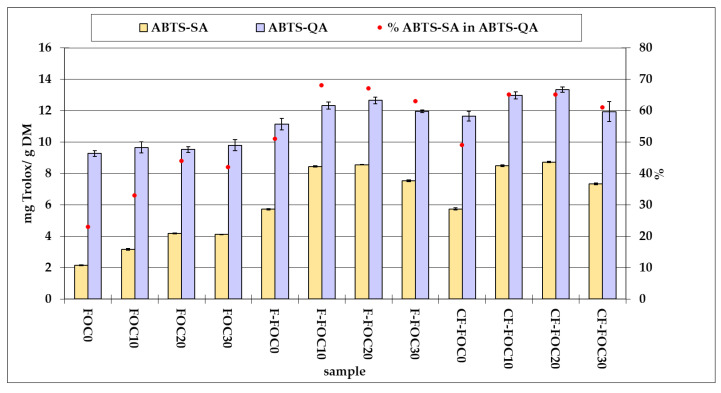
Effect of substrate kind and type of fermentation on ABTS˙^+^ scavenging activity. Data are shown as the mean ± SEM; FOC0—grass pea seeds, FOC10–FOC30—grass pea seeds with a 10–30% (*w*/*w*) flaxseed oil-cake addition; F—fermented; CF—co-fermented; ABTS-SA—ABTS˙^+^-scavenging assay; ABTS-QA—ABTS˙^+^-quencher assay.

The ability to neutralize ABTS^+•^ was measured by means of two methods. The quencher method (QA) allows estimating the total antiradical potential that results from the presence of both soluble and insoluble compounds in the material. The method applied to extracts (SA) captures the activity of the fraction soluble in a specific phase, here—in a neutral pH buffer. Fermentation resulted in an increased total ABTS^+•^ scavenging activity by 28% (Table 1). The activity of ABTS^+•^ neutralization by the soluble fraction was 123% higher in both fungal and fungal–bacterial fermentation products, as compared to the respective substrates. The magnitude of this change suggests that the metabolic activity of mycelium improved the solubility of antiradical components in the material. As previously shown, in legume seeds the ABTS^+•^ scavenging activity resulting from the presence of insoluble-bound phenols exceeds the antiradical potential of free phenolic compounds [13]. As observed for fermentation substrates, the ABTS^+•^-scavenging assay (SA) accounted for 23% (FOC0) to 44% (FOC20) of ABTS^+•^-quencher assay (QA), whereas for tempe—as much as 51% (FOC0) to 67–68% (FOC10 and FOC20) (Figure 1). The ABTS^+•^-SA was significantly influenced by the substrate kind (Table 1). Products obtained from FOC20 were characterised by the highest antiradical activity (8.6 mg Trolox/g DM), 50% higher than that of tempe made solely from grass pea seeds. Statistical analysis did not prove that bacterial fermentation increased the antiradical potential of the products, which confirms earlier findings according to which fermentation of legumes with *L. plantarum* does not necessarily result in the greater antiradical activity of the soluble fraction. The effect is largely dependent on the legume species [11].

#### 2.1.3. Hydroxyl Radical Scavenging Activity

The method applied in the experiment allows measuring the overall potential to both prevent the generation of ^•^OH in the Fenton reaction and its neutralization [14]. ^•^OH-SA depended on the substrate kind, being higher with the increase in oil-cake content up to 20%, and also on the fermentation method (Table 1). The mean effective dose (ED50) measured for fungal and fungal–bacterial fermentation products accounted for 41% and 38%, respectively, of ED50 of the substrates prepared for inoculation. Tempe obtained from FOC20 and FOC30 by means of co-fermentation were characterised by the highest ^•^OH-SA (ED50 accounted for 65% of the values measured for the FOC0 tempe).

### 2.2. The Profile of Phenolic Compounds

It is acknowledged that a solvent used for phenolic extraction strongly influences the amount and composition of the extracted compounds [15]. In this study, buffer with a neutral pH and low ionic strength was applied because all the antioxidant potential assessments were performed for aqueous extracts. Moreover, water-soluble compounds are most easily accessible during digestion.

The most common hydroxybenzoic acids were detected in the FOC0 substrate. The level of p-hydroxybenzoic acid and protocatechuic acid was 3 and 0.045 μg/g DM, respectively. Gallic acid was not found. The content of hydroxycinnamic acids ranged from about 3 to 0.3 μg/g DM for chlorogenic acid, and ferulic and sinapic acid, respectively (Table 2). The amounts determined in the extracts were considerably lower than those reported by Fratianni et al. [16]. However, it should be mentioned that grass pea seeds were subjected to soaking, dehulling and cooking prior to inoculation with microorganisms. Phenolic compounds in legume seeds are usually located in the hulls [17]. Water-soluble compounds may also have been washed from the seeds during soaking and boiling [17].

The addition of flaxseed oil-cake to grass pea seeds resulted in the appearance of gallic acid and an increase in its amount depending on the oil-cake dose, up to over 9 μg/g DM. The presence of oil-cake in the material also caused the accumulation of other phenolic acids. In FOC30, an increase from 6-fold (p-hydroxybenzoic acid) to 17-fold (vanillic acid) was observed. Flaxseed oil-cake did not influence the level of protocatechuic, syringic and chlorogenic acids in the material (Table 2). However, oil-cake was the only source of lignan—SECO, and its glycoside—SDG. In the chromatograms of the substrates and the tempe containing oil-cake, a peak with a retention time of 45.815 min and a spectrum corresponding to or very close to the spectrum of SDG and SECO was visible (Table 3). On this basis, it was identified as SDG oligomers. Lignan oligomers from flaxseed are composed of SDG and herbacetin diglucoside ester-linked by 3-hydroxy-3-methylglutaric acid and *p*-coumaric acid glucoside, as well as ferulic acid glucoside ester-linked to SDG [18]. The content of SDG oligomers in the fermentation substrates ranged from 216 to 422 μg/g DM, depending on the oil-cake share in the material.

Fungal fermentation resulted in a noticeable increase in gallic acid, but only in the case of material containing a maximum of 10% flaxseed oil-cake. The addition of *L. plantarum* to the inoculum caused further accumulation of gallic acid only in FOC0. The observed phenomenon could have resulted from the decomposition of hydrolysing tannins. It has been proven that moulds such as *Aspergillus* sp, *Penicillium* sp. and *Rhizopus oryzae* produce tannases [19]. Therefore, it is possible that *R. oligosporus* DSM 1967 applied in the present experiment also has such activity. The role of *L. plantarum* in the release of gallic acid from hydrolysing tannins has been confirmed in a different study [8].

Fungal fermentation also resulted in an increase in the protocatechuic acid (with the exception of FOC0) and *p*-hydroxybenzoic acid. Co-fermentation promoted the further accumulation of protocatechuic acid but only in materials that contained up to 10% flaxseed oil-cake. Tempe products obtained with mixed inoculum were characterised by a 45 to 116-fold higher level of protocatechuic acid, as compared to the values measured in FOC30 and FOC10 substrates, respectively. The change in the level of *p*-hydroxybenzoic acid observed after fermentation was by far the most pronounced (10-fold) in the case of FOC0 and FOC10. The level of the said compound was undoubtedly the highest among all phenolic acids determined in aqueous extracts, at an average of 32 μg/g DM in tempe. Protocatechuic and *p*-hydroxybenzoic acids can be a metabolite of both fungi and bacteria [20,21]. It has been proven that these compounds show antioxidant activity [22] and additionally protocatechuic acid is capable of up-regulation of antioxidant enzymes (e.g., glutathione peroxidase and glutathione reductase) expression [23]. With regard to hydroxycinnamic acids, fungal fermentation resulted in a significant decrease in the chlorogenic acid content, from 70% (FOC0) to more than 80% (FOC20), as well as a decrease in the content of ferulic and sinapic acid in products containing oil-cake, by 30% (FOC20) to 50% (FOC10 and FOC30). On the other hand, in tempe obtained after fermentation with *R. oligosporus* alone, more caffeic acid was determined, by about 60% (FOC20) to as much as 645% (FOC0). The decrease in chlorogenic acid observed after tempe fermentation indicates that *R. oligosporus* DSM 1964 showed esterase activity capable of hydrolysing this phenol. Slightly lower levels of chlorogenic acid (no statistically significant differences) measured in co-fermented material, as compared to fungal fermented one, might be caused by bacterial activity. Sánchez-Maldonado et al. [24] showed that *L. plantarum* metabolizes chlorogenic acid to caffeic acid to a small extent. When grass pea seeds alone were used as the fermentation substrate, fungal fermentation and co-fermentation resulted in an increase in the level of p-coumaric acid, by 25% and 47%, respectively.

The fermentation process generally had no effect on SECO and SDG levels in samples containing flaxseed oil-cake (Table 2). Nevertheless, the presence of these compounds in the aforementioned substrates and products could enhance their antioxidant potential, as compared to FOC0. Both SECO and SDG show antioxidant activity [6]. Fungal fermentation resulted in an increase in SDG oligomers only in the case of FOC20. The observed two-fold rise of SDG oligomers can only be explained by an increase in their extractability as a result of the metabolic activity of *R. oligosporus* mycelium that loosened cell structures of flaxseed oil-cake. It should be stressed that after the fermentation of this particular substrate, the highest glucosamine level and dry substance loss (indicators of mycelial growth/activity) were measured (data not shown).

Generally speaking, no clear impact of bacterial activity on the level of water-extractable phenols was observed. The ability of *Lactobacillus plantarum* to metabolize various phenols with the participation of such enzymes as phenolic acid decarboxylase and acid phenol reductase has been proven. However, the activity of these enzymes is inducible and depends on both the level of available phenols and the bacterial growth conditions [8].

### 2.3. Antioxidant Potential after In Vitro Digestion

Antioxidant potential was determined in dialysates obtained after in vitro digestion by the FCRS level, ABTS^+•^-SA and ^•^OH-SA.

The amount of FCRS released to dialysates depended on the substrate kind (Table 4). The highest level of the said compounds was measured in FOC10 and FOC20. Dialysates obtained from other substrates contained 12% less FCRS. The bioprocessing method also significantly affected this parameter. The digestion of fungal and fungal–bacterial fermented material resulted in the release of 21% and 35% more FCRS, respectively, as compared to the substrates. The highest amount of antioxidant compounds was determined in the case of dialysates obtained from co-fermented FOC10 and FOC20, on average, 40% more than in the case of grass pea tempe. The level of FCRS measured in dialysates after in vitro digestion was highly correlated with ABTS^+•^-SA (R^2^ = 0.92, *p* = 0.000). Therefore, it is not surprising that very similar changes to those described above were observed for ABTS^+•^ neutralization activity. The addition of 10% and 20% of flaxseed oil-cake increased the ABTS^+•^-SA of dialysates by 14%. At the same time, both fungal fermentation and co-fermentation resulted in higher scavenging potential of the samples, by 15% and 44%, respectively. The best source of compounds capable of ABTS^+•^ neutralization was tempe obtained after co-fermentation of FOC10 and FOC20.

The contents of FCRS and ABTS^+•^ scavenging compounds determined in dialysates can be compared to the results obtained for buffer extracts (Section 2.1.1–Section 2.1.2). In vitro digestion alone contributed to a pronounced increase in the antioxidant potential. The amount of FCRS in dialysates was 1.8 to 6.6-fold higher than the initial FCRS content measured in the materials, whereas the ABTS^+•^-SA was 3 to 10-fold higher. Digestive enzymes can promote the release of phenols from plant cells as well as phenols bound with other compounds [25]. The level of FCRS determined in dialysates was also significantly correlated with ^•^OH scavenging activity (R^2^ = 0.7, *p* = 0.000). The ^•^OH-SA depended on the substrate kind, although it was not directly related to the flaxseed oil-cake level because the highest values were determined for dialysates from FOC0 and FOC20 (Table 4). The potential of in vitro digestion products to scavenge ^•^OH depended to a greater extent on the bioprocessing method. Fungal fermentation and co-fermentation caused a 61% and 144% increase in the ^•^OH-SA, respectively, as compared to their substrates. The highest value (approximately 100 mg Trolox/g DM) was determined after the digestion of co-fermented FOC20. This sample was characterised by an ^•^OH-SA 60% higher than other co-fermented materials and about 110% higher than all fungal fermented products. It is worth mentioning again that tempe from FOC20 was the richest source of water-soluble SDG oligomers. Considering the structure of lignan macromolecule [18], it seems highly probable that they were present in dialysate and could neutralize hydroxyl radicals [6].

## 3. Materials and Methods

### 3.1. Fermentation Substrates

Grass pea (*Lathyrus sativus* L.) seeds cultivar Krab were obtained from “Spójnia” Hodowla i Nasiennictwo Ogrodnicze (Nochowo, Poland). Cold-pressed flaxseed oil-cake was kindly provided by Przedsiębiorstwo Nasienne CENTRALA NASIENNA Sp. z o.o. (Sanok, Poland).

### 3.2. Inoculum

Tempe strain *Rhizopus oligosporus* DSM 1962 (German Collection of Microorganism and Cell Cultures) was grown on potato extract agar. Spores were harvested after 12 days with a sterile saline solution (8 g/L) supplemented with peptone (0.01 g/L) and Tween 80 (0.1 mL/L). Next, the suspension was filtered three times (ϕ 11 μm, Nylon Net Filtres, Millipore, Cork, Ireland). The spore density was obtained by the spore-counting method in a Thoma chamber.

*Lactobacillus plantarum* DSM 20174 was rehydrated from a freeze-dried culture and grown at 30 °C on de Man, Rogosa and Sharpe (MRS) broth (BioMaxina S.A., Lublin, Poland) for 24 h. The bacterial cells were then centrifuged and suspended in a sterile saline solution. The cell density was measured by the turbidimetric method using McFarland’s standards.

### 3.3. Preparation of Flaxseed Oil-Cake

Flaxseed oil-cake was hydrated to 40% moisture content and simultaneously acidified to pH 4−5 with an appropriate quantity of 5% lactic acid, then sterilized (121 °C, 20 min) and cooled to room temperature.

### 3.4. Preparation of Seeds

Grass pea seeds were thoroughly cleaned and boiled in tap water for 30 min. Then, they were soaked in tap water for 18 h at room temperature. Next, the seeds were dehulled by hand and boiled for 15 min in tap water acidified to pH 4.5−5.0 with lactic acid. After discarding water, the seeds were drained and cooled (<35 °C).

### 3.5. Preparation of Fermented Products

Grass pea seeds alone or with the addition of flaxseed oil-cake were mixed thoroughly with the spore suspension of *R. oligosporus* (2 · 10^6^ spores per 100 g of raw material) or the inoculum containing *R. oligosporus* (2 · 10^6^ spores per 100 g of raw material) and *L. plantarum* (2 · 10^6^ cells per 100 g of raw material). The inoculated material was tightly packed in Petri dishes (ø 11 cm, four Petri dishes for each combination of substrate and fermentation inoculum) and incubated at 30 °C. Fermentation was stopped after 27 h by steaming the obtained products for 10 min. The tempe samples were lyophilized and stored at 4 °C for further analysis.

Eight types of grass pea-based-tempe were prepared after fungal and simultaneous fungal–bacterial fermentation of the following substrates: grass pea seeds (FOC0), and grass pea seeds with a 10%, 20% and 30% (*w*/*w*) flaxseed oil-cake addition (FOC10, FOC20 and FOC30, respectively).

### 3.6. Preparation of Pre-Treated Substrates

Grass pea seeds alone (FOC0), as well as grass pea seeds and flaxseed oil-cake mixtures (FOC10−30), subjected to the hydration and sterilization process (as described in Section 3.3 and Section 3.4), were lyophilized and kept at 4 °C for further analysis.

### 3.7. Buffer Extracts Preparation

Lyophilized material was used to prepare extracts at a concentration of 1 g/25 mL in a sodium-phosphate buffer (0.02 mol/L, pH 7.4). Extracts were used to measure substances reacting with Folin–Ciocalteu reagent (FCRS) and ^•^OH and ABTS^+•^ scavenging activity.

### 3.8. Analytical Methods

Dry matter (DM) was obtained with a moisture analyser (WPS 110S, Radwag, Radom, Poland) at 105 °C.

FCRS were assessed by the method of Swain and Hillis [26]. Briefly, 5 mL of properly diluted extracts was mixed with 0.25 mL of 1 mol/L Folin–Ciocalteu reagent and 0.5 mL of saturated Na_2_CO_3_ solution. After 15 min of incubation, absorbance of the samples was measured at 700 nm against the reagent blank. The result was expressed as mg gallic acid/g DM.

The hydroxyl radical (^•^OH) scavenging activity (^•^OH-SA) was estimated according to the assay given by Marambe et al. [27]. Briefly, 50–600 μL of extract was mixed with appropriate volume of potassium phosphate buffer (20 mmol/L, pH 7.4) to achieve 1125 μL of sample. Next, the following components were sequentially added: 40 μL of 0.5 mmol/L FeCl_3_, 42 μL of 2.4 mmol/L EDTA, 140 μL of 0.02 mol/L deoxyribose, 10 μL of 0.01 mol/L ascorbic acid and 142 μL of 1 mmol/L H_2_O_2_. The assay mixture was incubated at 37 °C for 1 h and after that 1 mL of 1% (*w*/*v*) TBA and 1 mL of 2.8% (*w*/*v*) TCA were added. The sample was incubated at 100 °C for 20 min, and after cooling to room temperature, the absorbance was measured at 532 nm against a blank. The blank consisted of all the reagents, to which TBA and TCA were added prior to incubation at 37 °C. The ^•^OH-SA was expressed as ED50 (effective dose—defined as mg of the sample used for the extraction that is required for the inhibition of 50% free radicals in the reaction conditions) or as mg Trolox equivalents/g DM.

The ABTS radical scavenging activity (ABTS^+•^-SA) was obtained as described by Arnao et al. [28]. To prepare the ABTS^+•^ solution, 10 mg of ABTS [2,20-azino-bis(3-ethylbenzothiazoline-6-sulfonic acid)] was dissolved in 1.3 mL of 0.0049 mol/L K_2_S_2_O_8_ and 1.3 mL of double distilled water, and left for 16 h at room temperature. Just before analysis, ABTS^+•^ solution was diluted with phosphate buffer to achieve the absorbance of 0.7 ± 0.02 at 734 nm. Next, 2 mL of ABTS^+•^ solution was mixed with 200 μL extract and incubated at room temperature in the dark. After 7 min, the absorbance was measured at 734 nm, with phosphate buffer as a reference. The ABTS^+•^ scavenging activity was expressed as mg Trolox equivalents/g DM.

Quencher-ABTS^+•^ assay (ABTS^+•^-QA) was performed according to Gökmen et al. [29]. Briefly, 10 mg of lyophilized material was mixed with 30 mL of ABTS^+•^ solution (initial absorbance 0.7 ± 0.02 at 734 nm), and shaken at room temperature in the dark. After 7 min, the absorbance of filtered samples was measured at 734 nm, with phosphate buffer as a reference. The ABTS^+•^ scavenging activity was expressed as mg Trolox equivalents/g DM.

### 3.9. Phenolic Profile

The profile of phenolic compounds extractable in buffer was estimated according to Lin et al. [30] with some modifications concerning the initial composition of mobile phase and time of analysis.

#### 3.9.1. LC-DAD Condition

The LC-DAD instrument consisted of an HPLC (Biorad Lab., Herkules, CA. USA) coupled with a diode array detector (DAD) and a column LUNA C18 (5 μm, 250 × 4.6 mm) was used at a flow rate of 1.0 mL/min. The column oven temperature was set at 25 °C. The mobile phase consisted of a combination of A (0.1% formic acid in water) and B (0.1% formic acid in acetonitrile). The gradient was varied linearly from 5% to 10% B (*v*/*v*) in 10 min, from 10% to 26% B (*v*/*v*) in 40 min, to 65% B in 80 min and finally to 100% B in 81 min and held at 100% B to 85 min. Finally, the column was equilibrated for 10 min. The DAD was set at 230, 257, 274, 310 and 350 nm for real-time read-out, and UV/VIS spectra, from 190 to 650 nm, were continuously collected.

#### 3.9.2. LC-DAD Condition

One gram of lyophilized material was extracted with 10 mL of phosphate buffer (0.02 mol/L, pH 7.4) for 3 h, centrifuged (10,000 rpm, 15 min) and then filtered through a 0.45 μm 13 mm Nylon syringe filter (FilterBio^®^ NY Syringe Filter, Labex Ltd., Nantong City, Jiangsu P.R, China). A 50 μL of the extract or standard (1 mg/100 mL of buffer) was injected into the column for analysis.

### 3.10. In Vitro Digestion

Lyophilized material was digested using the method with pepsin and pancreatin, as described in Stodolak et al. [31]. Briefly, 0.5 g of material with the addition of 1.7 mg of pepsin (Sigma, Steinheim, Germany, 4750 U/mg) dissolved in 0.1 mol/L HCl was incubated at 37 °C, pH 2.0 for 2 h. Then, 2.5 mg of pancreatin (Sigma, from porcine pancreas, 8× USP specifications) dissolved in 0.1 mol/L NaHCO_3_ was added and the sample was incubated at 30 °C, pH 7.0 for 4 h in dialysis tubes (Sigma Aldrich, cellulose membrane 25 mm × 16 mm, retaining most proteins with molecular weight ≥ 12,000) immersed in 50 mL of phosphate buffer (0.2 mol/L, pH 7.0). In dialysates (phosphate buffer with the compounds that passed the membrane), the level of FCRS (mg gallic acid/g DM) and activity against ABTS^+•^ and ^•^OH (mg Trolox equivalents/g DM) were estimated.

### 3.11. Statistical Analysis

For the in vitro digestion test, four samples were prepared, each analysed in two replications. For the other analyses four replications were made. The results were statistically evaluated using the two-way analysis of variance (with the exception of glucosamine and phenolic compounds profile—one-way ANOVA), where factor 1 was a treatment kind and factor 2 was a substrate kind. To determine statistically significant differences, the Tukey post-hoc test was used (*p* ≤ 0.05). To estimate correlation between parameters, regression analyses were done at *p* ≤ 0.05. Data were processed using Statistica version 12.0 software (StatSoft, Inc., Tulsa, OK, USA).

## 4. Conclusions

In this paper, we show that an addition of 20% flaxseed oil-cake and the application of bacterial–fungal co-fermentation, can be considered as an effective tool to improve the antioxidant potential of grass pea tempe. The co-fermented tempe was enriched in phenolic acids (gallic acid, protocatechuic acid) and had higher antioxidant activity than the fungal fermentation products. Moreover, after in vitro digestion, more antioxidant compounds were released from tempe obtained with the use of mixed inoculum than from the mould-fermented products.

## Figures and Tables

**Table 1:** Antioxidant activity of substrates and fermented products.

		FCRS (mg/g DM)	˙OH-SA (ED50)	ABTS˙^+^-SA (mg Trolox/g DM)	ABTS˙^+^-QA (mg Trolox/g DM)
**Treatment Kind × Substrate Kind**					
pre-treated	FOC0	1.44 ± 0.01 a	14.10 ± 0.27 h	2.14 ± 0.03 a	9.27 ± 0.18 a
	FOC10	2.23 ± 0.01 b	9.52 ± 0.11 g	3.17 ± 0.05 b	9.66 ± 0.34 ab
	FOC20	2.75 ± 0.00 c	5.33 ± 0.08 e	4.19 ± 0.03 c	9.52 ± 0.18 a
	FOC30	3.28 ± 0.01 d	6.84 ± 0.06 f	4.11 ± 0.02 c	9.80 ± 0.34 ab
fungal fermented	FOC0	3.40 ± 0.02 de	4.61 ± 0.06 d	5.72 ± 0.05 d	11.14 ± 0.37 bc
	FOC10	5.68 ± 0.05 f	3.77 ± 0.03 bc	8.44 ± 0.04 f	12.33 ± 0.22 cde
	FOC20	6.07 ± 0.06 g	3.50 ± 0.14 b	8.56 ± 0.01 g	12.65 ± 0.21 de
	FOC30	5.64 ± 0.06 f	2.79 ± 0.02 a	7.53 ± 0.05 e	11.95 ± 0.08 cde
co-fermented	FOC0	3.51 ± 0.06 e	4.22 ± 0.10 cd	5.74 ± 0.06 d	11.65 ± 0.30 cd
	FOC10	6.01 ± 0.02 g	3.71 ± 0.02 bc	8.49 ± 0.05 f	12.97 ± 0.22 de
	FOC20	6.75 ± 0.06 h	2.97 ± 0.02 a	8.73 ± 0.05 g	13.35 ± 0.17 e
	FOC30	5.59 ± 0.05 f	2.77 ± 0.02 a	7.34 ± 0.06 e	11.94 ± 0.63 cde
**Treatment Kind**					
pre-treated		2.41 ± 0.16 A	8.95 ± 0.70 C	3.39 ± 0.16 A	9.56 ± 0.13 A
fungal fermented		5.19 ± 0.26 B	3.66 ± 0.13 B	7.56 ± 0.24 B	12.01 ± 0.16 B
co-fermented		5.47 ± 0.30 C	3.42 ± 0.11 A	7.57 ± 0.25 B	12.48 ± 0.24 B
**Substrate Kind**					
FOC0		2.77 ± 0.29 A	7.65 ± 1.10 C	4.54 ± 0.40 A	10.68 ± 0.32 A
FOC10		4.64 ± 0.51 B	5.68 ± 0.67 B	6.69 ± 0.61 C	11.66 ± 0.40 B
FOC20		5.20 ± 0.53 D	3.92 ± 0.24 A	7.15 ± 0.50 D	11.84 ± 0.45 B
FOC30		4.83 ± 0.32 C	4.14 ± 0.45 A	6.33 ± 0.37 B	11.24 ± 0.34 AB

Two-way analysis of variance and Tuckey post-hoc test were applied (treatment kind—factor 1; substrate kind—factor 2; substrate kind × treatment kind—interaction between factors). Data are shown as the mean ± SEM. Mean values within a column followed by different letters differ significantly (*p* ≤ 0.05). FCRS—Folin–Ciocalteu reacting substances; ABTS˙^+^-SA—ABTS˙^+^-scavenging assay; OH˙-SA—OH˙-scavenging assay; ABTS˙^+^-QA—ABTS˙^+^-quencher assay; ED50—effective dose; substrates: FOC0—grass pea seeds, FOC10–FOC30—grass pea seeds with a 10–30% (*w*/*w*) flaxseed oil-cake addition.

**Table 2:** Profile of phenolic compounds in substrates and fermented products.

		Pre-treated	Fungal Fermented	Co-Fermented
**Gallic Acid (μg/g DM)**				
Substrate kind	FOC0	0.000 ± 0.000 aA	1.444 ± 0.485 bA	3.691 ± 0.065 cA
	FOC10	1.100 ± 0.023 aA	4.320 ± 0.588 bA	3.939 ± 0.036 bA
	FOC20	5.606 ± 0.395 aB	4.291 ± 0.794 aA	5.337 ± 0.240 aB
	FOC30	9.362 ± 0.210 bC	3.248 ± 0.008 aA	3.368 ± 0.492 aA
**Protocatechuic Acid (μg/g DM)**				
Substrate kind	FOC0	0.045 ± 0.014 aA	0.079 ± 0.010 aA	4.173 ± 0.104 bB
	FOC10	0.050 ± 0.006 aA	3.058 ± 0.098 bB	5.799 ± 0.404 cC
	FOC20	0.050 ± 0.015 aA	4.118 ± 0.048 bC	4.320 ± 0.122 bB
	FOC30	0.059 ± 0.003 aA	2.640 ± 0.204 bB	2.657 ± 0.246 bA
***p*-Hydroxybenzoic Acid (μg/g DM)**				
Substrate kind	FOC0	2.972 ± 0.639 aA	30.984 ± 1.218 cA	27.124 ± 0.315 bA
	FOC10	2.933 ± 0.567 aA	31.813 ± 1.933 bA	31.892 ± 0.631 bB
	FOC20	14.651 ± 1.714 aB	30.278 ± 1.915 bA	26.073 ± 1.563 bA
	FOC30	18.731 ± 0.035 aC	40.220 ± 0.991 bB	41.189 ± 1.337 bC
**Vanillic Acid (μg/g DM)**				
Substrate kind	FOC0	0.161 ± 0.053 aA	1.454 ± 0.239 bA	1.169 ± 0.064 bA
	FOC10	0.767 ± 0.125 aAB	3.437 ± 0.674 aAB	3.924 ± 1.316 aA
	FOC20	1.914 ± 0.041 aBC	1.968 ± 0.165 aAB	2.452 ± 0.630 aA
	FOC30	2.813 ± 0.471 aC	3.802 ± 0.320 aB	4.780 ± 0.042 aA
**Syringic Acid (μg/g DM)**				
Substrate kind	FOC0	1.488 ± 0.045	1.336 ± 0.023	1.570 ± 0.132
	FOC10	1.378 ± 0.156	1.541 ± 0.040	1.163 ± 0.249
	FOC20	1.045 ± 0.092	0.976 ± 0.188	0.821 ± 0.316
	FOC30	1.004 ± 0.032	0.814 ± 0.246	2.244 ± 0.648
**Chlorogenic Acid (μg/g DM**)				
Substrate kind	FOC0	2.886 ± 0.081 bA	1.054 ± 0.278 aA	0.726 ± 0.081 aA
	FOC10	3.501 ± 0.716 bA	0.882 ± 0.031 aA	0.602 ± 0.048 aA
	FOC20	2.446 ± 0.063 bA	0.492 ± 0.016 aA	0.341 ± 0.077 aA
	FOC30	2.203 ± 0.152 bA	0.875 ± 0.096 aA	0.594 ± 0.069 aA
**Caffeic Acid (μg/g DM)**				
Substrate kind	FOC0	0.426 ± 0.031 aA	3.176 ± 0.056 cA	2.716 ± 0.001 bA
	FOC10	1.191 ± 0.040 aB	3.184 ± 0.102 bA	2.904 ± 0.348 bA
	FOC20	2.427 ± 0.098 aC	3.842 ± 0.105 bB	3.957 ± 0.191 bB
	FOC30	3.895 ± 0.267 aD	4.786 ± 0.179 abC	5.702 ± 0.197 bC
***p*-Coumaric Acid (μg/g DM)**				
Substrate kind	FOC0	0.541 ± 0.018 aA	0.680 ± 0.021 bA	0.798 ± 0.032 cA
	FOC10	1.908 ± 0.038 bB	1.668 ± 0.041 bB	1.069 ± 0.169 aA
	FOC20	2.617 ± 0.057 bC	2.184 ± 0.049 aC	2.192 ± 0.090 aB
	FOC30	3.639 ± 0.157 bD	2.565 ± 0.200 aD	3.360 ± 0.055 bC
**Ferulic & Sinapic Acid (μg/g DM)**				
Substrate kind	FOC0	0.338 ± 0.044 aA	0.239 ± 0.009 aA	0.169 ± 0.036 aA
	FOC10	1.977 ± 0.275 bB	0.993 ± 0.004 aB	0.688 ± 0.147 aB
	FOC20	2.750 ± 0.072 bC	1.933 ± 0.102 aC	1.899 ± 0.020 aC
	FOC30	3.857 ± 0.168 cD	1.959 ± 0.166 aC	2.849 ± 0.098 bD
**Secoisolariciresinol (SECO) (μg/g DM)**				
Substrate kind	FOC0	0.00 A	0.00 A	0.00 A
	FOC10	0.609 ± 0.042 aAB	0.440 ± 0.015 aAB	0.386 ± 0.121 aA
	FOC20	0.748 ± 0.036 aAB	0.870 ± 0.009 bBC	0.669 ± 0.006 aA
	FOC30	1.305 ± 0.399 aB	2.230 ± 0.348 aC	1.322 ± 0.479 aA
**Secoisolariciresinol Diglucoside (SDG) (μg/g DM)**				
Substrate kind	FOC0	0.00 A	0.00 A	0.00 A
	FOC10	2.569 ± 0.053 aB	2.679 ± 0.373 aB	2.645 ± 0.282 aB
	FOC20	6.759 ± 0.226 aC	6.124 ± 0.235 aC	6.345 ± 0.118 aB
	FOC30	12.942 ± 0.013 bD	8.736 ± 1.178 aD	10.417 ± 0.021 abD
**Secoisolariciresinol Diglucoside Oligomers (SDG Oligomers) (μg/g DM)**				
Substrate kind	FOC0	0.00 A	0.00 A	0.00 A
	FOC10	216.108 ± 14.154 aB	191.644 ± 4.805 aB	249.658 ± 8.340 aB
	FOC20	340.146 ± 39.628 aBC	687.715 ± 22.626 bC	787.046 ± 7.599 bC
	FOC30	422.054 ± 83.111 aC	310.644 ± 21.656 aD	275.049 ± 25.153 aB
**Sum (μg/g DM)**				
Substrate kind	FOC0	8.919 ± 0.816 aA	40.547 ± 1.598 bA	42.220 ± 0.187 bA
	FOC10	234.458 ± 14.405 aB	246.029 ± 6.972 aB	305.107 ± 7.092 bB
	FOC20	381.978 ± 40.944 aBC	745.862 ± 24.884 bC	842.627 ± 8.865 bC
	FOC30	482.879 ± 83.162 aC	384.191 ± 22.043 aD	355.804 ± 28.112 aB

For each phenolic compound, one-way ANOVA was applied. Data are shown as the mean ± SEM Mean values within a column followed by different big letters differ significantly (*p* ≤ 0.05). Mean values within a row followed by different small letters differ significantly (*p* ≤ 0.05). Substrates: FOC0—grass pea seeds, FOC10–FOC30—grass pea seeds with a 10–30% (*w*/*w*) flaxseed oil-cake addition.

**Table 3:** Retention time and absorption wavelengths of determined phenolic compounds by HPLC-DAD method.

Compound	Retention Time (min)	Absorption Max (nm)	Absorption Min (nm)
Gallic acid	7.9	271.8; 215.3	240.46
Protocatechuic acid	14.457	294.3; 259.5; 204.6	282.0; 236.0
Chlorogenic acid	11.445	327.4; 247.2; 197.9	264.0
*p*-Hydroxybenzoic acid	12.6	256.1; 194.9	236.0
Vanillic acid	14.51	292.1; 261.8; 201.2	281.95; 234.9
Caffeic acid	14.93	323.2; 215.7; 193.4	262.8
Syringic acid	15.07	274.1; 215.8	241.6
SDG	19.5	280.1; 197.9	253.9
Coniferyl alcohol	20.35	264.0; 241.6	211.0
*p*-Coumaric acid	22.11	309.6; 223.5; 207.9	249.2; 217.9
Sinapic acid	24.7	323.5; 199.9	263.9
Ferulic acid	24.8	322.3; 212.4; 194.5	261.0
SECO	33.96	282.0; 197.7	254.9
SDG oligomers	45.815	285.3; 195.6	240.5

**Table 4:** Antioxidant activity of substrates and fermented products after in vitro digestion.

		FCRS (mg/g DM)	ABTS˙^+^-SA (mgTrolox/g DM)	˙OH-SA (mgTrolox/g DM)
**Treatment Kind x Substrate Kind**				
pre-treated	FOC0	9.52 ± 0.04 bc	22.66 ± 0.30 abc	46.02 ± 3.69 bcd
	FOC10	8.77 ± 0.14 ab	22.02 ± 0.35 ab	34.23 ± 1.87 b
	FOC20	8.18 ± 0.23 a	20.89 ± 0.60 b	19.04 ± 1.02 a
	FOC30	8.30 ± 0.22 a	20.85 ± 0.19 ab	15.98 ± 0.84 a
fungal fermented	FOC0	9.96 ± 0.05 cd	24.42 ± 0.39 cd	49.24 ± 2.00 cde
	FOC10	10.97 ± 0.06 e	26.00 ± 0.37 d	40.50 ± 3.12 bc
	FOC20	10.91 ± 0.21 de	25.80 ± 0.26 d	47.01 ± 2.00 cd
	FOC30	10.10 ± 0.07 cde	23.03 ± 0.49 bc	49.74 ± 0.78 cde
co-fermented	FOC0	9.56 ± 0.10 bc	24.28 ± 0.25 cd	61.33 ± 2.39 e
	FOC10	13.30 ± 0.25 f	34.56 ± 0.50 f	59.08 ± 2.89 de
	FOC20	13.23 ± 0.40 f	34.96 ± 0.74 f	97.43 ± 4.76 f
	FOC30	10.62 ± 0.18 de	30.16 ± 0.47 e	59.82 ± 0.77 e
**Treatment Kind**				
pre-treated		8.63 ± 0.13 A	21.55 ± 0.23 A	28.81 ± 2.71 A
fungal fermented		10.45 ± 0.10 B	24.78 ± 0.28 B	46.48 ± 1.29 B
co-fermented		11.67 ± 0.31 C	31.00 ± 0.80 C	70.26 ± 3.90 C
**Substrate Kind**				
FOC0		9.69 ± 0.05 A	23.88 ± 0.23 A	51.40 ± 2.05 B
FOC10		11.01 ± 0.40 B	27.54 ± 1.11 B	43.42 ± 2.76 A
FOC20		10.77 ± 0.49 B	27.15 ± 1.21 B	54.49 ± 8.04 B
FOC30		9.66 ± 0.23 A	24.77 ± 0.85 A	42.27 ± 4.33 A

Two-way analysis of variance and Tuckey post-hoc test were applied (treatment kind—factor 1; substrate kind—factor 2; substrate kind × treatment kind—interaction between factors). Data are shown as the mean ± SEM. Mean values within a column followed by different letters differ significantly (*p* ≤ 0.05). FCRS—Folin–Ciocalteu reacting substances; ABTS˙^+^-SA—ABTS˙^+^-scavenging assay; OH˙-SA—OH˙-scavenging assay; substrates: FOC0—grass pea seeds, FOC10–FOC30—grass pea seeds with a 10%–30% (*w*/*w*) flaxseed oil-cake addition.
